# HER2 amplification as a resistance mechanism to osimertinib and its successful treatment with trastuzumab deruxtecan: a case report

**DOI:** 10.3389/fonc.2026.1921556

**Published:** 2026-09-23

**Authors:** Mansi Sharma, Ritwika Choudhury, Abhinav Dewan, Sabeena K. Choudhary, Priyanka Venkatraman, Sunil Pasricha, Anurag Mehta, Kundan Chufal, LM Darlong, Ullas Batra

**Affiliations:** 1 Department of Medical Oncology, Rajiv Gandhi Cancer Institute & Research Centre, New Delhi, India; 2 Department of Research (Medical Oncology Unit 5), Rajiv Gandhi Cancer Institute & Research Centre, New Delhi, India; 3 Department of Histopathology, Rajiv Gandhi Cancer Institute and Research Centre, New Delhi, India; 4 Department of Laboratory, Molecular Diagnosis and Transfusion Services, Rajiv Gandhi Cancer Institute & Research Centre, New Delhi, India; 5 Department of Radiation Oncology, Rajiv Gandhi Cancer Institute and Research Centre, New Delhi, India; 6 Department of Thoracic Oncosurgery, Rajiv Gandhi Cancer Institute and Research Centre, New Delhi, India

**Keywords:** case report, HER2 amplification, NSCLC, osimertinib resistance, trastuzumab deruxtecan (T-DXd)

## Abstract

Acquired resistance to osimertinib in EGFR-mutant advanced non-small cell lung cancer is inevitable, and management following progression remains an evolving clinical challenge. HER2 amplification is a recognised but underreported off-target resistance mechanism, and its optimal management is not yet established. Trastuzumab deruxtecan has shown activity in HER2-altered NSCLC, but real-world data describing its use in this specific resistance context, particularly with respect to intracranial activity, remain scarce. We report the case of a 50-year-old man with metastatic NSCLC harbouring an EGFR exon 19 deletion who experienced disease progression through multiple lines of therapy, including 3 years of first-line osimertinib, platinum-based chemotherapy, atezolizumab-bevacizumab-paclitaxel-carboplatin, and repeated radiotherapy for intracranial metastases. Comprehensive genomic profiling at progression identified acquired HER2 (ERBB2) amplification, confirmed by immunohistochemistry (IHC 3+) and fluorescence *in situ* hybridisation, alongside persistence of the original EGFR mutation. Following a deliberate washout period to mitigate interstitial lung disease risk, the patient received trastuzumab deruxtecan, a HER2-directed antibody-drug conjugate, as fifth-line therapy, achieving a systemic partial response sustained over seven cycles, with concurrent regression of intracranial lesions. This case underscores HER2 amplification as a clinically actionable resistance mechanism and demonstrates T-DXd’s systemic and intracranial activity in this setting. It also highlights the value of serial molecular profiling at each point of progression in guiding personalised treatment strategies while acknowledging that the optimal testing strategy requires prospective validation.

## 1 Introduction

Osimertinib is the current mainstay of treatment for epidermal growth factor receptor *(EGFR)*–mutant advanced non-small cell lung cancer (NSCLC), either as monotherapy or in combination (1, 2). However, acquired resistance is inevitable, and only a small proportion of patients on osimertinib monotherapy maintain durable responses beyond 18–24 months, with both on-target and off-target mechanisms contributing to disease progression (3, 4). Among off-target mechanisms, human epidermal growth factor receptor 2 (HER2) amplification represents a recognised bypass pathway that activates alternative signalling cascades (5). HER2 amplification has been reported in approximately 2% of patients after first-line osimertinib and up to 5% in the AURA study (6, 7). Similarly, the role of HER2 overexpression as a targetable-resistance mechanism is evolving (8, 9). The therapeutic approach following osimertinib resistance is complex and evolving, with no universally accepted standard treatment currently available (10). We present a case report demonstrating clinically meaningful disease control with Trastuzumab deruxtecan (T-DXd) in a patient with *EGFR*-mutant NSCLC who developed acquired HER2 amplification after first-line osimertinib and achieved long-term survival through use of sequential therapies. We present the following case in accordance with the CARE reporting checklist.

## 2 Case presentation

### 2.1 Initial diagnosis and treatment

A 50-year-old male presented with complaints of productive cough and generalised weakness for three months. He had no addictions or comorbidities. There was no relevant family or psychosocial history, and no known hereditary cancer predisposition. On examination, his vitals, general and systemic examinations were unremarkable.

Imaging with chest radiography and contrast-enhanced CT of the chest revealed a well-defined mass in the left lower lobe (~49.3 mm × 41 mm × 30.4 mm), multiple bilateral pulmonary nodules and few small lymph nodes in the carinal and left hilar regions. Contrast-enhanced brain MRI revealed no enhancing parenchymal or meningeal lesions.

CT-guided biopsy with immunohistochemistry (IHC) confirmed moderately differentiated adenocarcinoma NSCLC. Molecular profiling using next-generation sequencing (NGS) revealed an *EGFR* exon 19 deletion and *TP53* baseline mutation. The patient received osimertinib 80 mg daily for 3 years with significant clinical and radiological response.

### 2.2 Disease progression on osimertinib and subsequent therapy

He developed progression with cytology-positive pleural effusion, and NGS revealed persistence of the original baseline mutations. The patient received systemic chemotherapy consisting of four cycles of pemetrexed (500 mg/m^2^) and carboplatin (AUC 5) followed by eleven cycles of maintenance pemetrexed over a period of one year. Biopsy done from the new liver lesion on progression did not reveal any small cell transformation. He received two courses of FSRT (fractionated stereotactic radiation therapy) to the new brain lesions (27 Gy in 3 fractions each), administered three months apart (**Figure 1**), followed by the atezolizumab, bevacizumab, paclitaxel, and carboplatin (ABCP) regimen at standard doses (pending NGS report) and had a partial response after three cycles.

**Figure 1:**
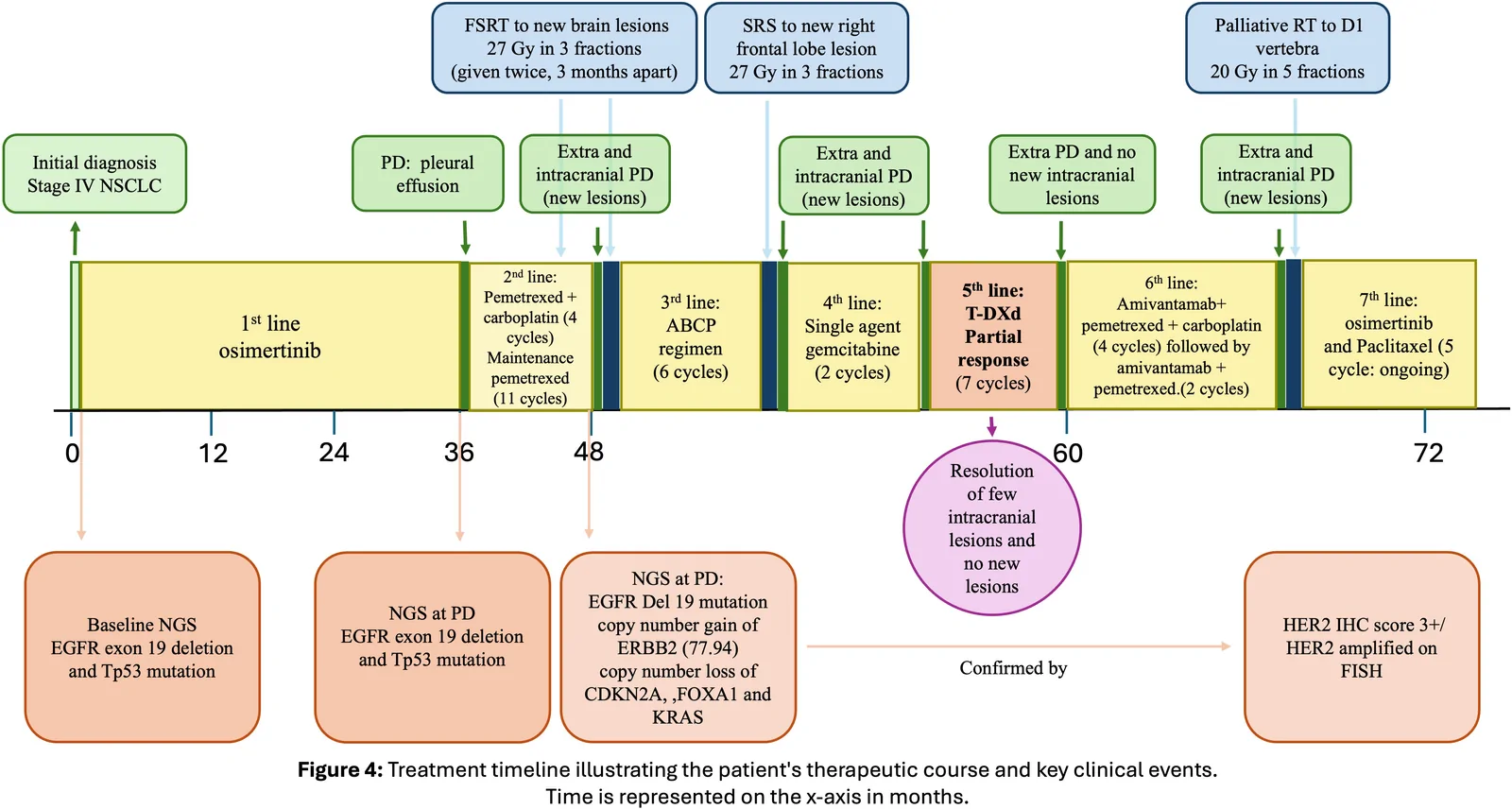
Treatment timeline illustrating the patient's therapeutic course and key clinical events. Time is represented on the x-axis in months.

### 2.3 Molecular profiling and detection of HER2 alteration

Subsequently, comprehensive genomic profiling revealed the original *EGFR* Del 19 mutation, copy number gain of *ERBB2*: 77.94 (Erb-B2 Receptor Tyrosine Kinase 2), and other multiple alterations, namely, copy number loss of *CDKN2A*, *FOXA1*, and *KRAS*. HER2 receptor scoring by IHC demonstrated diffuse and strong membranous staining (IHC 3+), with 100% of tumour cells showing complete membranous positivity, and HER2 was additionally amplified on fluorescence *in-situ* hybridisation (FISH) analysis (**Figure 2**) (**Table 1**) (15). Given that the patient had demonstrated a partial response to ABCP, the decision was made to complete the planned treatment course before transitioning to HER2-directed therapy.

**Figure 2:**
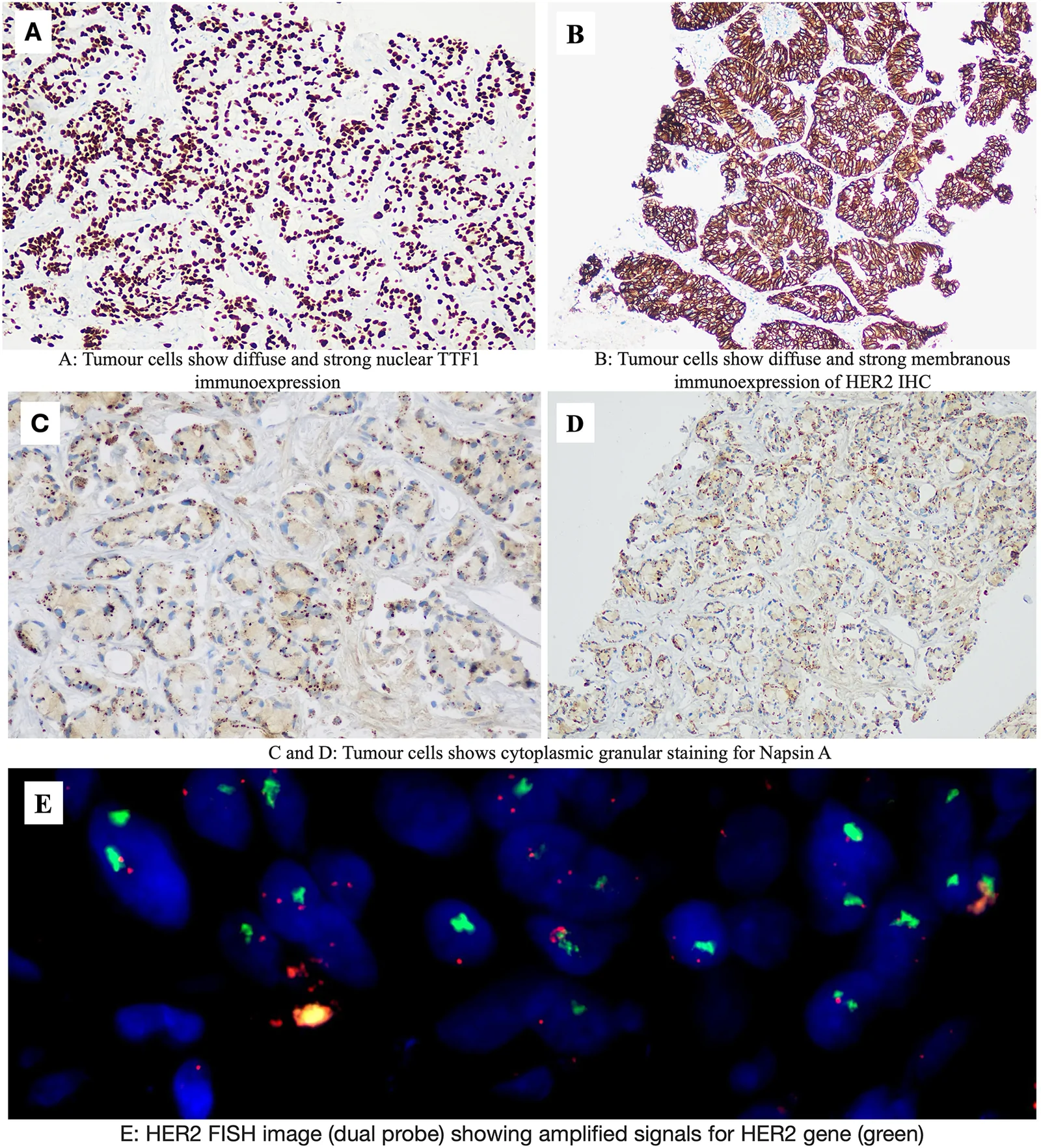
Pathological findings: Histopathological examination of the biopsy specimen This image is published with the patient's consent.

**Table 1:** Summary of published cases of anti-HER2 therapy in NSCLC with acquired HER2 alteration.

Cases	Sharma et al., 2026 (Present case)	Ge et al., 2026, 11	Odintsov et al., 2024, 12	McMahon et al., 2026, 13	Hirata et al., 2025, 14
Primary mutation	*EGFR* exon 19 deletion	*EGFR* G719X (exon 18, uncommon) at baseline; L858R on re-biopsy	Not reported individually	*ERBB2*-mutant NSCLC (exon 20 duplication; Y772_A775dup)	*EGFR* exon 19 deletion
1st-line therapy	Osimertinib (3 years)	Afatinib (1st-line metastatic)	Osimertinib	Carboplatin + pemetrexed	Osimertinib
Acquired HER2 alteration type	*ERBB2* amplification (CN 77.94)	HER2 amplification	HER2 amplification	*ERBB2* amplification (CN > 8)	*HER2* exon 20 insertion (A775_G776insYVMA) (*HER2* mutation)
Molecular method used at progression to determine resistance pathway	Tissue NGS + IHC 3+ (100% of cells) + FISH	Tissue NGS (~20 copies)	NGS + FISH	NGS + IHC	Liquid biopsy (cfDNA) + tissue NGS
CNS disease at anti-HER2 initiation	Yes Multiple pre-existing brain metastases (post FSRT ×2 and SRS)	No New brain metastasis developed during T-DXd	Not reported individually	Yes Prior brain metastases; intracranial data not available as a measured endpoint	Not reported
Anti-HER2 agent and line of treatment	T-DXd (5th line)	T-DXd (4th line)	Anti-HER2 ADC (clinical trial; agent not specified); line not reported individually	T-DXd (5th line)	T-DXd + osimertinib (combination) (4th line)
Dose received	5.4 mg/kg (full standard dose)	4.4 mg/kg (dose-reduced)	Not reported	Not reported	5.4 mg/kg (starting); 4.4 mg/kg (tolerated dose)
Duration of extracranial response	Partial response ~5 months (7 cycles)	Partial response ~2 months	One patient: 11 months on anti-HER2 ADC (individual response data not disaggregated)	Partial response >8 months (ongoing at last follow-up)	Partial response ~8 months
Duration of intracranial response on anti-HER2 agent	Regression of pre-existing brain metastases, no new intracranial lesions for ~10 months from T-DXd initiation	New cerebellar metastasis (disease progression) at ~4 months	Not reported individually	Not reported as a measured endpoint	Disease progression at ~8 months
Status at last follow-up	Alive, ongoing 7th-line therapy ~6 years from Stage IV diagnosis	Deceased (~7 months after T-DXd initiation)	Not reported individually	Alive Maintaining response on T-DXd (January 2026)	Deceased

### 2.4 T-DXd treatment and outcome

Following disease progression after six cycles of ABCP, the patient received a third course of FSRT to the new brain lesions, followed by single-agent gemcitabine 1 g/m^2^ (Day 1, Day 8, and Day 15 schedule) for two cycles. This interval was a deliberate clinical decision to mitigate interstitial lung disease (ILD) risk, reflecting an active safety calculation rather than a delay in targeted therapy. Brain MRI demonstrated further progression of intracranial lesions, with concurrent extracranial disease progression involving pleural deposits, lymph nodes, liver, and bone.

T-DXd was initiated as a fifth-line treatment at the dose of 5.4 mg/kg every 3 weeks. A partial response to treatment was observed after three cycles, with radiologic regression of intracranial and extracranial lesions (**Figures 3**, **4**) (**Table 1**). He received seven cycles over 5 months with no significant toxicity: no evidence of ILD on high-resolution computed tomography (HRCT) chest assessment performed every two cycles and no reduction in ejection fraction. Further evaluation demonstrated extracranial disease progression, and brain MRI revealed an increase in the size of some brain lesions.

**Figure 3:**
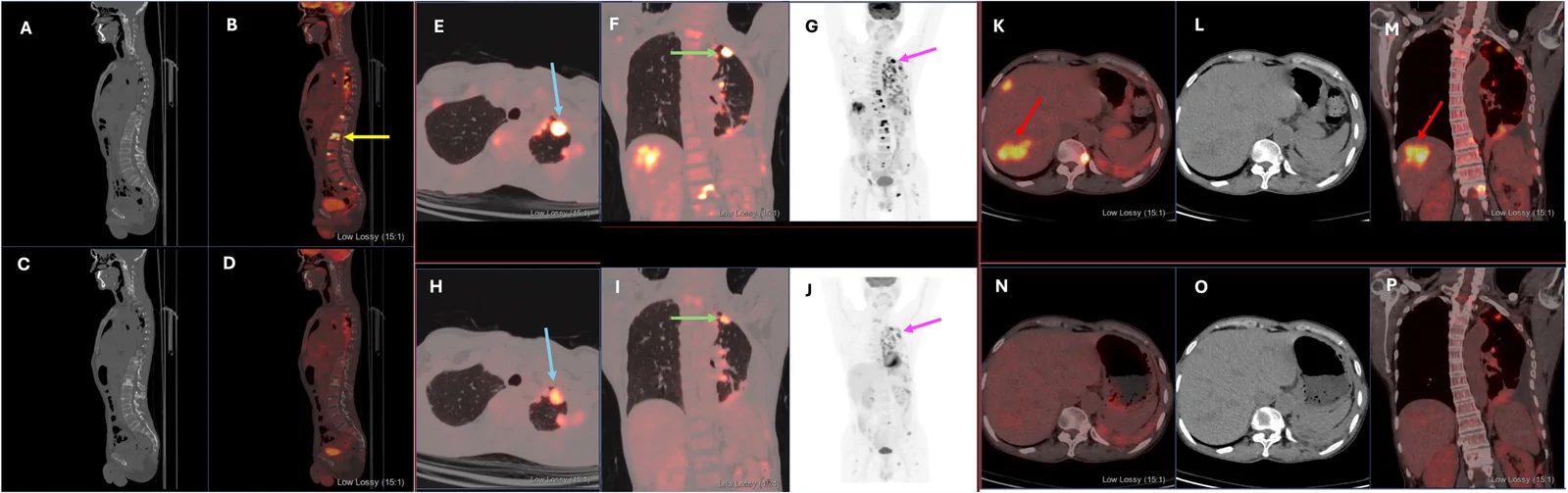
18F FDG PET CT scan was done 60 mins after injection of 222MBq of FDG and the images were reconstructed with standard TOF reconstruction parameters to generate transaxial, coronal and sagittal images. Sagittal images demonstrating multiple metabolically active lytic and sclerotic vertebral lesions before treatment **(A, B)**. Post-treatment images of the same region show marked regression of disease with increased sclerosis, consistent with a healing response **(C, D)**. Transverse **(E)** and coronal thoracic **(F, G)** images showing left lung lesions at baseline and post treatment regression in metabolic activity (SUV max) and size suggesting partial response to treatment **(H–J)**. Transverse **(K, L)** and coronal **(M)** images showing multiple metabolically active liver lesions before treatment and significant regression following treatment **(N–P)**. This image is published with the patient's consent.

**Figure 4:**
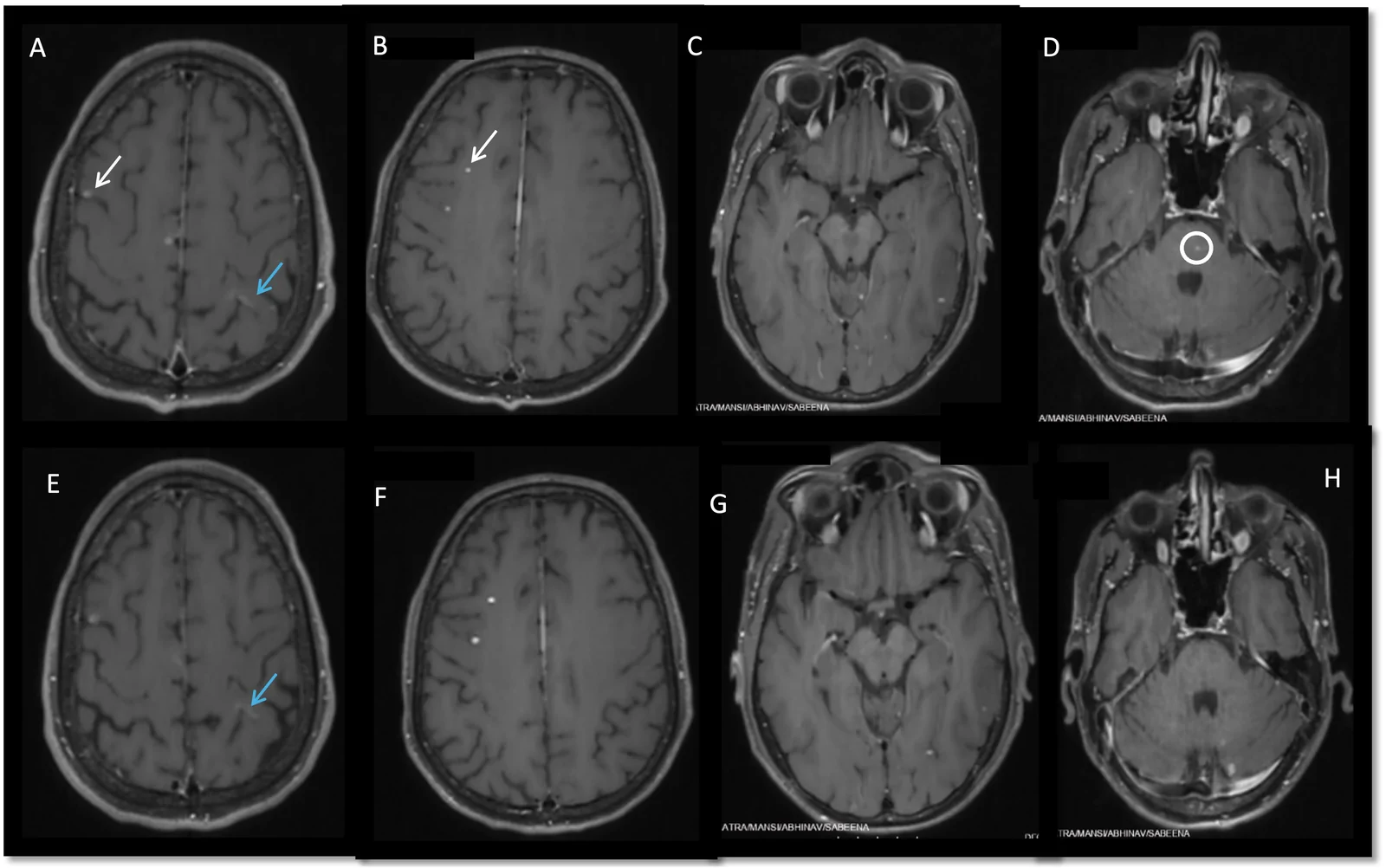
CEMRI brain in patient of CA lung on T-DXd. **(A-D)** demonstrate scan of patient before treatment and **(E-H)** demonstrate follow up scan (post treatment). Multiple punctate foci of hyperenhancemement noted in B/L cerebral hemispheres, pons and left cerebellar hemisphere (white arrow). Few of the foci are seen to resolve on follow up scan (white circles),with some lesions appearing stable in size. **(A, E)** show focal curvilinear gyral enhancement in post central gyrus (blue arrow) - persistent on follow up scan. This image is published with the patient's consent.

### 2.5 Post T-DXd treatment

The patient was started on the sixth line of treatment with amivantamab, pemetrexed, and carboplatin (four cycles), followed by two cycles of amivantamab and pemetrexed. A brain MRI was repeated after three months and showed no further intracranial progression until nearly five months after discontinuation of T-DXd. Upon systemic disease progression, he received palliative radiotherapy (RT) to the D1 vertebra, following which he was initiated on seventh-line treatment with osimertinib and paclitaxel (five cycles up to date), which is ongoing.

## 3 Discussion

### 3.1 Osimertinib resistance mechanisms

Acquired resistance to osimertinib is broadly categorised into *EGFR*-dependent (“on-target”) mechanisms and *EGFR*-independent (“off-target”) mechanisms (5). Common mechanisms include *MET* amplification and *EGFR* C797S mutation, while others comprise histological transformation and HER2 alterations.

### 3.2 Biological significance of the HER-family signalling in acquired *EGFR* TKI resistance

The *EGFR* tyrosine kinase family comprises four transmembrane proteins with similar structures: ERBB1 (*EGFR*, HER1), *ERBB2* (HER2), ERBB3 (HER3), and ERBB4 (HER4) (16, 17). HER2 possesses intrinsic protein tyrosine kinase activity that activates downstream signalling pathways involved in cell growth and survival. Unlike other members of the HER family, it does not bind to any specific ligand and is activated primarily through heterodimerisation with other member proteins to mostly activate the ERK pathways (17).

In contrast, HER3 possesses very low intrinsic kinase activity and activates downstream PI3K/AKT pathway signalling upon heterodimerisation with receptors such as HER2, *EGFR*, and MET, thereby promoting cellular growth and survival (18–21). The HER2–HER3 heterodimer could potentially contribute to osimertinib resistance by activating all available HER2 and HER3 receptors and multiple pathways and thus bypass *EGFR* inhibition.

This receptor dimerisation is frequently enhanced during *EGFR* TKI (tyrosine kinase inhibitor) treatment and may contribute to the development of TKI resistance (20). Preclinical studies have shown that osimertinib upregulates cell surface HER2 expression (22, 23). Additionally, increased HER3/HER2 heterodimer formation has been associated with reduced response to *EGFR* TKIs (24).

### 3.3 HER2 alterations as acquired resistance mechanisms

HER2 alterations in NSCLC are categorised into HER2 gene mutations, HER2 protein overexpression and HER2 gene amplification (25). HER2 mutations as resistance mechanisms after osimertinib are rare, and case reports suggest potential efficacy of HER2-directed antibody-drug conjugates (ADCs) such as T-DXd in this setting (12, 14) (**Table 1**).

HER2 protein overexpression is typically assessed using IHC and may occur independently of gene amplification, where it may be linked to polysomy (25, 26). Unlike in breast cancer, where HER2 IHC overexpression closely parallels gene amplification, the relationship between IHC and FISH in NSCLC is substantially weaker (8). Protein overexpression without underlying gene amplification is common in lung tumours, and only 10%–20% of HER2 IHC 3+ NSCLC cases demonstrate *ERBB2* amplification on FISH (**Figure 2**) (8).

HER2 amplification results from an increased HER2 gene copy number and is seen in ~1%–2% of post-first-line osimertinib biopsy samples (5).

### 3.4 HER2 amplification as a resistance mechanism in our case

In our case, FISH analysis demonstrated true HER2 gene amplification rather than chromosome 17 polysomy or polyploidy. The average HER2 signal count was 15 copies per nucleus, while the centromere enumeration probe for chromosome 17 (CEP17) showed an average of three copies per nucleus, resulting in a HER2/CEP17 ratio of 5.0 (15/3). In cases of chromosome 17 polysomy or polyploidy, proportional increases in both HER2 and CEP17 copy numbers would be expected, yielding a ratio close to 1.0 (27). In contrast, the markedly elevated ratio observed in our case reflects selective amplification of the HER2 gene relative to chromosome 17, confirming genuine HER2 amplification as the underlying molecular alteration.

The co-existing alterations on comprehensive genomic profiling included copy number loss of *CDKN2A*, *FOXA1*, and *KRAS*, which reflects the genomic complexity of a heavily pre-treated tumour; however, high-level *ERBB2* copy number gain (CN 77.94), diffuse HER2 IHC 3+ staining, and FISH-confirmed amplification support HER2 amplification as the dominant clonal event at progression. The robust systemic and intracranial response to T-DXd, without concurrent *EGFR*-targeted therapy, may also indicate that HER2 may have become the predominant oncogene causing resistance to osimertinib, further supported by the fact that baseline molecular testing had not demonstrated this alteration.

### 3.5 Role of T-DXd in overcoming *EGFR* TKI resistance

T-DXd is a HER2 ADC and consists of a humanised anti-HER2 IgG1 monoclonal antibody with a tetrapeptide-based cleavable linker and a membrane-permeable topoisomerase I inhibitor payload. T-DXd uses modern ADC design principles embodying the principle of linker fitness, which balances high plasma stability with tumour-selective payload release, which distinguishes it from earlier-generation ADCs.

It has a drug-to-antibody ratio of eight payload molecules per antibody. Its cleavable linker is designed to favour intracellular payload release following internalization into HER2-expressing tumour cells, releasing the membrane-permeable DXd payload, which can then diffuse into adjacent tumour cells, extending cytotoxic activity through a bystander effect (28). It has been approved by the FDA in April 2024 for patients with advanced HER2 IHC 3+-expressing tumours who have had prior treatment with no viable options remaining, and present guidelines for NSCLC recommend the same in later lines of therapy (29).

T-DXd monotherapy has demonstrated clinically meaningful activity in HER2-overexpressing NSCLC in DESTINY-Lung03 Arm 1D (ORR 44.4%), and nearly half of the patients had received prior *EGFR*-targeted therapies. Pre-treatment biopsy confirmation of HER2 amplification was not a protocol-mandated requirement in DESTINY-Lung03. Similarly, an ORR (Overall Response Rate) of 34% was seen in Cohort IA in the DESTINY-Lung01 trial, which also included patients who had received previous *EGFR* TKIs (30).

Odintsov et al. (12) reported three cases with acquired HER2 amplification as a resistance mechanism to osimertinib, which was detected on NGS and confirmed via FISH, and one patient received anti-HER2 ADC in a clinical trial for 11 months (**Table 1**).

Another case report by Ge et al. (11) described ERBB2 amplification (~20 copies by tissue NGS) following afatinib and second-line osimertinib in G719X/L858R-positive disease, with subsequent response to T-DXd. The authors reported new brain metastasis as a disease progression event after 4 months of T-DXd. In contrast, our patient received first-line osimertinib for EGFR exon 19 deletion, the most commonly encountered *EGFR* mutation, representing a more clinically applicable scenario for this resistance mechanism. Another key distinction in our patient is that he achieved radiological regression of pre-existing intracranial lesions after starting T-DXd (**Figure 4**) (**Table 1**).

Our patient had developed multiple new intracranial lesions despite systemic therapy and brain FSRT over the 6 months preceding T-DXd initiation. Following a third course of FSRT for newly developing lesions, he was treated with gemcitabine but experienced further intracranial disease progression. No further radiation was given, and he developed regression of brain lesions at the first assessment after the initiation of T-DXd. Subsequently, the patient did not develop any new lesions for nearly 10 months from T-DXd initiation and had no requirement for further local CNS-directed therapy to date (14 months from T-DXd initiation). This indicates the drug’s CNS efficacy and may suggest a potential radiation-sparing strategy for selected patients with brain metastases.

Brain metastases represent a major unresolved challenge in EGFR-mutant NSCLC and exert a disproportionate impact on quality of life and survival. Wilcox et al. (31) reported that among 262 patients treated with first-line osimertinib, 53% developed CNS metastases, including 36% with *de novo* CNS disease and 16% developed CNS progression during treatment. The CNS activity of T-DXd has previously been demonstrated in the ERBB2-mutant setting, where Jänne et al. (32) showed intracranial responses in patients with active brain metastases across randomised trial populations; however, its role in the ERBB2-amplified setting is still being established.

A recently published case report by McMahon et al. (13) described an acquired ERBB2 amplification as a resistance mechanism to zongertinib (HER2-directed TKI) in a patient with primary ERBB2-mutant NSCLC, with a subsequent response to T-DXd (**Table 1**). Whilst this further supports real-world use of T-DXd in acquired HER2 amplification, it is mechanistically distinct from our case. The index targeted agent was a HER2-directed rather than EGFR-directed TKI, and the underlying molecular context was a primary HER2 mutation rather than acquired HER2 amplification following EGFR-directed therapy.

### 3.6 Therapeutic considerations in our case

Our patient developed HER2 amplification, resulting in HER2 overexpression, as an acquired resistance mechanism to osimertinib. He received seven cycles of T-DXd with sustained systemic disease control (**Figures 3**, **4**) and no significant toxicity. We did consider combination therapy with osimertinib and T-DXd, given persistence of the original *EGFR* mutation on repeat biopsy: this strategy has demonstrated efficacy in patients with acquired HER2 mutations following osimertinib resistance (14). However, concerns regarding the risk of lung injury limited this approach in our case.

Given the ILD rates reported with DESTINY-Lung01 IA (5%–20%) and DESTINY-Lung03 (5.6%), appropriate precautions were implemented to ensure our patient’s safety (8, 30). This included an HRCT chest at baseline and at regular intervals, delayed initiation of T-DXd post-immunotherapy, and use of 5.4 mg/kg dosing (associated with lower ILD rates) (30).

Although no consensus currently exists on optimal HER2 testing methodology in NSCLC, we used all three modalities concurrently to confirm the biological significance of HER2 amplification. HER2 expression may change over the course of disease (8, 9), making reassessment by IHC at progression a rapid and practical strategy for identifying patients who may benefit from T-DXd. If confirmed in larger prospective studies, HER2 IHC could become an important component of routine diagnostic workflows. Based on our experience with this single patient, we suggest that HER2 alteration testing should be done at the time of progression on osimertinib. Whether NGS, IHC, or FISH is optimal as a standalone test, and the degree of concordance between these three modalities in the post-osimertinib setting, remains to be established by larger prospective cohorts.

### 3.7 Strengths and limitations

A major strength of our approach was the comprehensive assessment of HER2 status in both pre- and post-progression biopsy specimens, providing an important distinction from previous reports (11, 12) and offering real-world evidence of T-DXd activity in HER2-amplified, HER2 IHC 3+ disease following first-line osimertinib (**Table 1**).

We performed repeated biopsies with serial molecular profiling at each stage of disease progression, enabling treatment decisions to be guided by evolving molecular alterations. This personalised approach is supported by previous evidence demonstrating improved survival outcomes among patients who receive targeted therapy directed at acquired alterations compared with those who do not undergo such molecularly guided treatment (33).

Notably, our heavily pre-treated patient demonstrated a concurrent intracranial response (**Figure 4**), possibly extending the signal of CNS activity from HER2-mutant NSCLC to the acquired HER2-amplified setting, for which dedicated intracranial response data are absent from existing trial datasets (32).

However, our study is limited to a single case, and more real-world data are required to replicate our findings.

## 4 Conclusion

T-DXd monotherapy demonstrated clinically meaningful systemic response and intracranial stability in a patient with *EGFR*-mutant NSCLC who developed acquired HER2 amplification following first-line osimertinib. The patient was treated with multiple lines and remains alive nearly six years after a Stage IV diagnosis. This case also highlights the impact of molecular profiling in identifying actionable targets at resistance. The optimal strategy for identifying HER2 alterations following osimertinib is yet to be defined, and further evidence is needed to understand the sequencing of T-DXd either alone or in combination in this patient population.

## Data Availability

The original contributions presented in the study are included in the article/**Supplementary Material**. Further inquiries can be directed to the corresponding author/s.
