# Supplementary material for: HER2 amplification as a resistance mechanism to osimertinib and its successful treatment with trastuzumab deruxtecan: a case report
Source: Front Oncol. 2026 Sep 23;16:1921556. doi: 10.3389/fonc.2026.1921556 (PMC13642157; doi:10.3389/fonc.2026.1921556)
Supplement: Supplementary file 1 [file Table1.docx]

**CARE Checklist of information to include when writing a case report**

*Manuscript: HER2 amplification as a resistance mechanism to osimertinib and its successful treatment with Trastuzumab Deruxtecan: A case report*

*Based on the 2013 CARE Checklist (care-statement.org/checklist).*

| **Item** | **Topic** | **Item Description** | **Reported in Manuscript** |
| --- | --- | --- | --- |
| **1** | Title | The diagnosis or intervention of primary focus followed by the words “case report”. | Title page |
| **2** | Key Words | 2 to 5 key words that identify diagnoses or interventions in this case report (including “case report”). | Key Words, p.1 (main manuscript, below Abstract) |
| **3a** | Abstract – Introduction | What is unique about this case and what does it add to the scientific literature? | Abstract: Background |
| **3b** | Abstract – Case | The patient’s main concerns and important clinical findings. | Abstract: Case Description |
| **3c** | Abstract – Case | The primary diagnoses, interventions, and outcomes. | Abstract: Case Description |
| **3d** | Abstract – Conclusion | What are one or more “take-away” lessons from this case report? | Abstract: Conclusions |
| **4** | Introduction | Briefly summarises why this case is unique and may include medical literature references. | Section 1) Introduction |
| **5a** | Patient Information | De-identified patient specific information. | Section 2.1) Initial Diagnosis and Treatment |
| **5b** | Patient Information | Primary concerns and symptoms of the patient. | Section 2.1) Initial Diagnosis and Treatment |
| **5c** | Patient Information | Medical, family, and psychosocial history including relevant genetic information. | Section 2.1) Initial Diagnosis and Treatment |
| **5d** | Patient Information | Relevant past interventions and their outcomes. | Sections 2.1–2.4 |
| **6** | Clinical Findings | Describe significant physical examination (PE) and important clinical findings. | Section 2.1) Initial Diagnosis and Treatment |
| **7** | Timeline | Historical and current information from this episode of care organised as a timeline (figure or table). | Figure 4: Treatment timeline |
| **8a** | Diagnostic Assessment | Diagnostic methods (PE, laboratory testing, imaging, surveys). | Sections 2.1, 2.3; Figures 1, 2, 3 |
| **8b** | Diagnostic Assessment | Diagnostic challenges. | Section 3.5) Strengths and limitations; Discussion on HER2 IHC/FISH discordance |
| **8c** | Diagnostic Assessment | Diagnosis (including other diagnoses considered). | Sections 2.1, 2.3 |
| **8d** | Diagnostic Assessment | Prognostic characteristics when applicable. | Section 3.2–3.3) HER2 Alterations / Role of T-DXd |
| **9a** | Therapeutic Intervention | Types of therapeutic intervention (pharmacologic, surgical, preventive). | Sections 2.1–2.4 |
| **9b** | Therapeutic Intervention | Administration of therapeutic intervention (dosage, strength, duration). | Section 2.4) Subsequent Treatment and Outcome |
| **9c** | Therapeutic Intervention | Changes in therapeutic interventions with explanations. | Section 2.4 (gemcitabine washout rationale; ILD risk mitigation) |
| **10a** | Follow-up and Outcomes | Clinician- and patient-assessed outcomes if available. | Section 2.4) Subsequent Treatment and Outcome |
| **10b** | Follow-up and Outcomes | Important follow-up diagnostic and other test results. | Section 2.4; Figures 2, 3 |
| **10c** | Follow-up and Outcomes | Intervention adherence and tolerability. (How was this assessed?) | Section 2.4 (HRCT chest assessment every two cycles, ejection fraction monitoring) |
| **10d** | Follow-up and Outcomes | Adverse and unanticipated events. | Section 2.4 (no significant toxicity reported) |
| **11a** | Discussion | Strengths and limitations in your approach to this case. | Section 3.5) Strengths and limitations |
| **11b** | Discussion | Discussion of the relevant medical literature. | Sections 3.1–3.4 |
| **11c** | Discussion | The rationale for your conclusions. | Section 3.4) Therapeutic Considerations; Conclusion |
| **11d** | Discussion | The primary “take-away” lessons from this case report (without references) in a one paragraph conclusion. | Section 4) Conclusion |
| **12** | Patient Perspective | The patient should share their perspective on the treatment(s) they received. | Patient declined to provide a perspective statement |
| **13** | Informed Consent | The patient should give informed consent. (Provide if requested.) | Section 2.4 (consent statement); Footnote: Ethical Statement |
